# Editorial: Consumer's Behavior Beyond Self-Report

**DOI:** 10.3389/fpsyg.2021.770079

**Published:** 2021-10-04

**Authors:** Alexandra Wolf, Kazuo Ueda

**Affiliations:** ^1^Department of Human Science, Research Center for Applied Perceptual Science, Kyushu University, Fukuoka, Japan; ^2^Unit of Perceptual Psychology, Department of Human Science/Research Center for Applied Perceptual Science/Division of Auditory and Visual Perception Research, Research and Development Center for Five-Sense Devices, Kyushu University, Fukuoka, Japan

**Keywords:** neuroscience, consumer behavior, physiology, marketing, application, eye-tracking, decision-making

## Introduction

Incorporating facial electromyography (fEMG), electrooculography (EOG), electroencephalography (EEG), electrodermal activity (EDA; also known as galvanic skin response), electrocardiography (ECG), and eye-tracking into scientifically valid experimental paradigms empower scientists to answer questions about how individuals perceive, manipulate, and use the information to complete a task (Bettiga et al.; Choi et al.; Hu and Shi; Kaneko et al.; Klichowski and Kroliczak; Kwon and Kim; Ounjai et al.; Wolf et al., 2018, 2019). Notably, the data obtained through the advanced techniques not only give valuable insights into one's attentional and perceptional processes or emotional arousal, but also reveal responses that consumers do not want or are not able to express, e.g., specific groups of consumers like patients suffering from mental disorders or children (Cherubino et al., 2019; Rojas et al.; Wolf and Ueda).

Popular data collection methods involve questionnaires, surveys, and interviews (i.e., self-report measures) to have an insight into consumers' decision-making process (e.g., product evaluation, willingness to purchase). The general acceptance, practicality, and low cost make self-reports popular; however, they are often described with issues of over- or underestimated recall (e.g., inaccurate memory), response bias, and the inability to capture consumers' unconscious reactions (Bell et al., 2018; Bettiga et al.; Yang et al.). It has been reported that prior studies regarding the discrimination between hedonic and utilitarian products are grounded on self-reported experiences, which assess conscious emotions that subjects can recognize and verbalize, but not unconscious feelings (i.e., happening without individual awareness) (Bettiga et al.). The work of Bettiga et al. presents physiological analyses and depicts consumers' unconscious affective reactions as powerful drivers of decision-making. In addition, the team provides an initial step toward using physiological data regarding the subjects' cardiac activity (ECG), respiratory activity, and electrodermal activity (EDA) to evaluate consumers' experience with new products. This contribution casts new light on the conventional discrimination between hedonic (linked to sensory satisfaction, pleasure, and excitement) and utilitarian products that are associated with more functional and practical benefits (Bettiga et al.).

Kaneko et al. also recognized changes in ECG and EDA as a reflection of emotional state during a food-tasting event. In addition, the research group used explicit and implicit measures (e.g., a modified willingness-to-take-home scale) to examine the effect of emotional state on the experience of a novel soup (traditional Japanese soup based on seaweed broth) vs. a familiar food product (vegetable soup). In this study, one group of participants faced a “positive emotion induction procedure” (i.e., a promise of a reward after tasting). In contrast, a modified Sing-a-Song Stress Test (which causes profound social stress) was used to induce a negative emotional state in the second group. In conclusion, both soups were experienced as equally pleasant in the positive emotion condition, while in the adverse emotion condition, the new product and the familiar soup were experienced as relatively unpleasant and relatively pleasant, respectively. Thus, presented findings show that emotional state affects food pleasantness differently for novel and familiar foods (Kaneko et al.). As for straightforward application, this work states that one should introduce a novel food when consumers are not stressed. Otherwise, it may negatively affect food pleasantness, with the negative effect remaining at least 1 week. Hence, a positive recommendation would be to let consumers taste a new product when they are in a positive mood (Kaneko et al.). Finally, an interesting note regarding electrodermal activity was made by Lajante et al. who reported that neuromarketing agencies often use smartwatches to insight individuals' arousal levels. However, his perspective article reveals important implications, that is, Lajante et al. underline that the evaluation of advertisements mainly relies not on arousal but pleasure. Therefore, special attention must be paid to the merits of the experimental method, where facial EMG might be more informative than electrodermal activity for measuring aesthetic emotions in advertising research (Lajante et al.).

**Figure 1:**
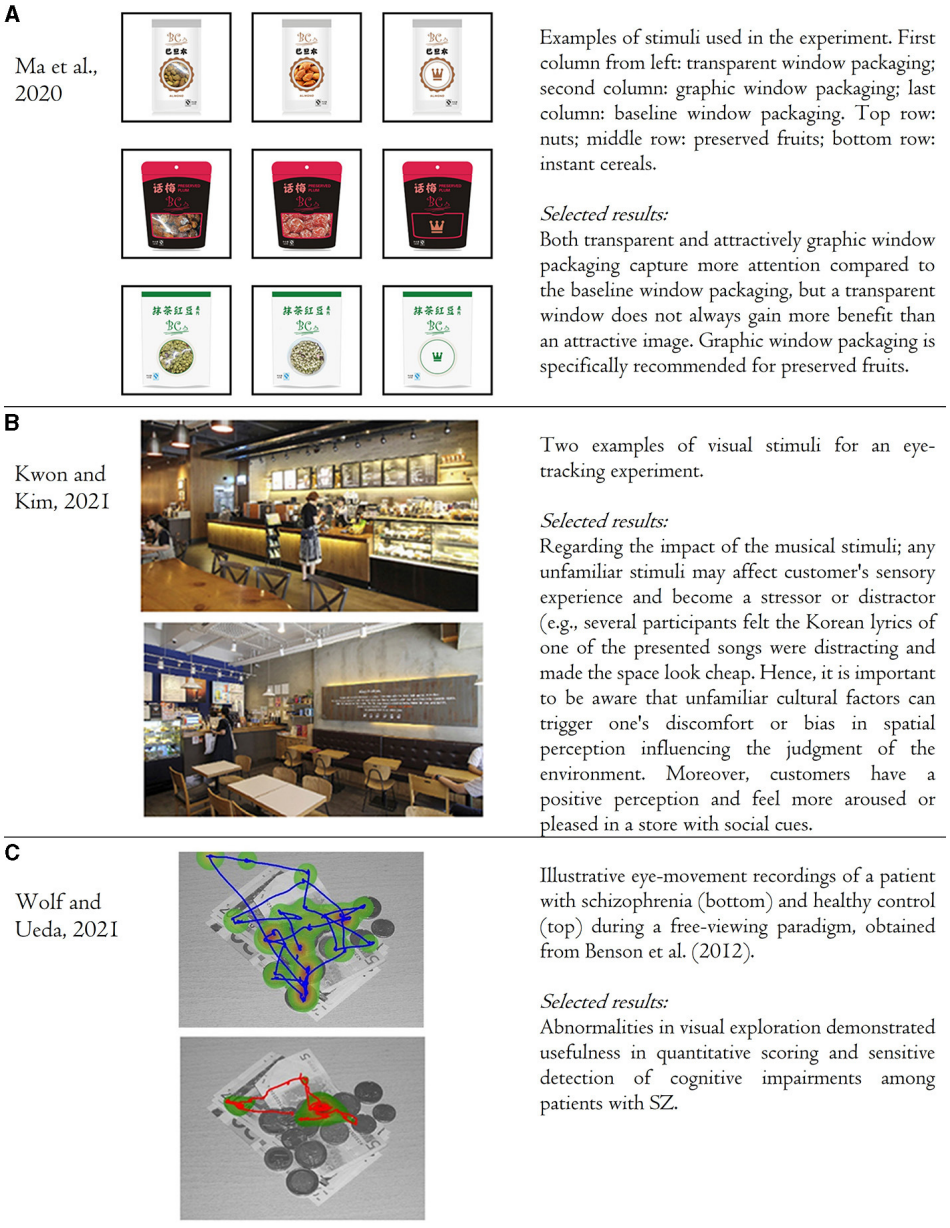
Application of eye-tracking technology in product packaging **(A)**, design of commercial stores **(B)**, and detection of cognitive impairments among clinical populations **(C)**.

In the last years, research efforts related to consumer science have used eye-tracking to elucidate individuals' visual processing of presented stimuli (Ounjai et al.; van der Laan et al., 2015; Wolf et al., 2018, 2019). Since consumers are usually not aware of the steps of simplifying their decision-making processes (i.e., ignoring some options and paying more attention to preferred alternatives), gaze behavior, where the slightest change in gaze allocation reflects a shift in information-prioritization, permits better identification of consumers' unconscious processes (Bialkova et al., 2020; Rojas et al.). For example, Ma et al. examined whether food packaging with a transparent window has more advantages in capturing consumer attention and determining consumers' willingness to purchase than packaging with a graphic window (at the same region and size of the package). With eye-tracking technology, the research group provided objective evidence on attentional capture of three different packaging types (kindly refer to Figure 1A). Also, the authors stated direct applications; for example, in order to design a visually attractive product that will enhance consumers' willingness to purchase, food manufacturers should consider the category of packed food (Ma et al.).

Implementing research findings can become standard practice not only in product development but in interior design as well (Spence, 2020). Interiors such as healthcare and children's environments have started actively adopting sensory design features based on scientific suggestions (Cox et al., 2004; Collier and Jakob, 2017; Cheng et al., 2019). However, similar information has not been available for retail design, even though retail settings often involve many sensory information channels, being an exciting environment for multi-sensory studies. Kwon and Kim provide information about what customers see in commercial stores (coffee shops) and what factors trigger their gaze (see Figure 1B). Through this work, the authors (1) suggest a methodological framework that encompasses qualitative as well as quantitative measures, (2) take a unique opportunity to discuss faced challenges, and (3) share implications for optimal use of eye-tracking technology in the discipline of interior design (Kwon and Kim).

The editors find it necessary to acknowledge that the scientific community primarily focused on experimental paradigms under controlled laboratory environments when the call for potential contributions was made. In order to raise the question as to whether real-life context experiments help explain consumers' decision-making strategy (i.e., beyond the experimental room), the editors addressed scientists who show interest in ecologically valid experiments. For example, the study conducted by Klichowski and Kroliczak presents unique findings regarding the most critical skill behind consumer behavior, which is the ability to assess whether a price after a discount is a real bargain. While there is an agreement that the left posterior parietal cortex (PPC) contributes to mental arithmetic, it is unknown if it is involved in calculations of sale prices. Therefore, the researchers examined the role of PPC in mental shopping calculations. Moreover, the group re-modeled their laboratory to resemble a shop and asked participants to calculate the product's price after discount. The findings of this study shed preliminary light on the topic of mental calculations in a natural setting (Klichowski and Kroliczak).

Concerning the context of a real market, Suomala presented a theoretical explanation of consumer behavior. His plausible framework of the Consumer Contextual Decision-Making Model concludes that, though the content of meaningfulness is different among individuals from different cultural backgrounds, the concept of meaningfulness is the most vital trigger of consumer choice. Moreover, he underlines that decision-making reflects subjective meaningfulness based on experiences rather than objective features of the physical world (Suomala). This suggests that the role of the consumer's mental system in a decision-making situation is to process given information successfully to achieve assigned or self-set goals. According to that, Yu et al. demonstrated that consumption could be biased by incidental mental status. Some consumers exhibit a great interest in symbolic products that highlight their values in the context of social status, intelligence, and lifestyle (i.e., to reconstruct their self-identity).

Interestingly, even subtle hints in advertisements might trigger a compensatory consumption (i.e., when individuals purchase a product, not for its functionality but its signaling value) (Yu et al.). Individuals' efforts to avoid the psychological discomfort of self-threats via such compensatory consumption can backfire with negative consequences (e.g., physical, financial, and psychological) and lead to long-term harm (Yu et al.). For instance, purchasing products that compensate for psychological deficits might drive consumers to spend money beyond their budget on products they usually would not buy or could not afford. Also, consuming food to regulate one's emotional distress might lead to the development of eating pathologies and obesity. Yang et al. reported that in comparison with healthy controls, patients with seasonal affective disorder (SAD) exhibit a higher frequency of hyperphagia (an increased desire for food), external eating (eating in response to external cues, such as sight or smell of food), and emotional eating (eating for reasons other than hunger) (Yang et al.).

Yet, there is insufficient research in the clinical domain that examines the decision-making processes among consumers, who suffer from psychiatric and mood disorders (Wolf et al., 2021). Wolf and Ueda underline that consumer neuroscience (that enriches understanding of consumer psychology and behavior) and neuroeconomics (that refers to sensemaking of economic problems through the analysis of neural correlates of decision making) should be studied, among healthy controls and patients, who suffer from mental disorders. Prominently, Wolf and Ueda put forward the aspects of combining eye-tracking methodology and real-life decision-making paradigms to disclose valuable information regarding patient's behavior and identify gaze metrics (such as scan-path length, see: Figure 1C) as potential biomarkers to improve diagnostic precision.

## Conclusions

The selection of contributions supports the statement that neurophysiological tools can highlight the mechanisms underpinned human behavior, and therefore lead to an improved understanding of consumers' thoughts, intentions, and believes that accompany the decision-making situation (Bettiga et al.; Choi et al.; Hu and Shi; Kaneko et al.; Kwon and Kim; Ounjai et al.; Wolf et al., 2018, 2019). Particularly, data captured by eye-trackers give valuable insights into how a viewer determines the subjective hierarchy of provided information and undertakes the decision strategy (Choi et al.; Danner et al., 2016; Motoki et al., 2021; Wolf and Ueda). Moreover, since self-reported and psychophysiological measures are more complementary than mutually exclusive, neuroscientific tools can increase the precision of self-report measures and give a more solid background to formulate future psychological laws and contribute to the broader discussion about driving forces in consumer behavior (Hu and Shi; Lajante et al.). Indeed, the Research Topic comprises studies that represent data from a relatively small number of participants. Nevertheless, gathered scientific contributions should be considered an inspiration for future paradigms that will reveal information about individuals' behavior at the population level (amongst healthy and clinical populations) and provide more explicit applications for marketing strategies.

While ethical implications in consumer research need to be continuously perfected (Wolf and Ueda), the Topic Editors express their hopes that the results of future replicable and non-invasive experimental paradigms, which record a range of physiological and neuroscientific data, will (1) improve and inform marketing strategies, (2) provide new frameworks for the explanation of consumer behavior in real market contexts, and last but not least (3) significantly support the knowledge regarding cognitive deficits among clinical populations.

## Author Contributions

AW wrote the manuscript with valuable revision from KU. All authors contributed to the article and approved the submitted version.

## Conflict of Interest

The authors declare that the research was conducted in the absence of any commercial or financial relationships that could be construed as a potential conflict of interest.

## Publisher's Note

All claims expressed in this article are solely those of the authors and do not necessarily represent those of their affiliated organizations, or those of the publisher, the editors and the reviewers. Any product that may be evaluated in this article, or claim that may be made by its manufacturer, is not guaranteed or endorsed by the publisher.
